# Evaluation of a Granular Bone Substitute for Bone Regeneration Using an Optimized In Vivo Alveolar Cleft Model

**DOI:** 10.3390/bioengineering10091035

**Published:** 2023-09-02

**Authors:** Alban Destrez, Emilien Colin, Sylvie Testelin, Bernard Devauchelle, Stéphanie Dakpé, Marie Naudot

**Affiliations:** 1 UR 7516 CHIMERE, University of Picardie Jules Verne, Chemin du Thil, CS 52501, 80025 Amiens, France; destrez.alban@chu-amiens.fr (A.D.); testelin.sylvie@chu-amiens.fr (S.T.); devauchelle.bernard@chu-amiens.fr (B.D.); dakpe.stephanie@chu-amiens.fr (S.D.); naudotm@gmail.com (M.N.); 2 Maxillofacial Surgery Department, Amiens University Hospital, Rond-point du Pr Christian Cabrol, 80054 Amiens, France; 3 Institut Faire Faces, Rond-point du Pr Christian Cabrol, 80054 Amiens, France

**Keywords:** alveolar cleft, biphasic calcium granules, bone regeneration, bone substitute, in vivo model, mesenchymal stem cells

## Abstract

Alveolar cleft is a common congenital deformity that requires surgical intervention, notably using autologous bone grafts in young children. Bone substitutes, in combination with mesenchymal stem cells (MSCs), have shown promise in the repair of these defects. This study aimed to evaluate the regenerative capabilities of a granular bone substitute using an optimized alveolar cleft model. Thirty-six rats underwent a surgical procedure for the creation of a defect filled with a fragment of silicone. After 5 weeks, the silicone was removed and the biomaterial, with or without Wharton’s jelly MSCs, was put into the defect, except for the control group. The rats underwent μCT scans immediately and after 4 and 8 weeks. Analyses showed a statistically significant improvement in bone regeneration in the two treatment groups compared with control at weeks 4 and 8, both for bone volume (94.64% ± 10.71% and 91.33% ± 13.30%, vs. 76.09% ± 7.99%) and mineral density (96.13% ± 24.19% and 93.01% ± 27.04%, vs. 51.64% ± 16.51%), but without having fully healed. This study validates our optimized alveolar cleft model in rats, but further work is needed to allow for the use of this granular bone substitute in the treatment of bone defects.

## 1. Introduction

The surgical management of facial pathologies often includes a reconstruction phase. The sequelae of cancer surgery, trauma and congenital malformations are all indications, but still represent a challenge given the variability of tissues (bone, muscle, nerve, etc.), the objectives, and the surgical procedures involved [1]. In the case of facial bone defects, autologous grafting is generally indicated and has long been performed, for example, by harvesting iliac crest, fibula, or calvarial bone from the parietal area, depending on the pathology [2,3,4]. However, the morbidity at the donor site and the quantity of available bone are limiting factors [5].

Orofacial clefts are among the most common congenital deformities, affecting approximately 1/700 births worldwide, with high ethnic and geographical variations [6]. South America tends to be one of the most-affected regions with more than 2 births out of 1000 being affected; among the least affected is Africa, with an average of 0.5/1000, and Western Europe, with an average of around 1.5/1000, and 1/1000 births in France [7]. Also, boys are more frequently affected, with a ratio of 2:1. The condition is characterized by a gap in the maxilla, which can extend to the palate and disrupt the alignment of teeth, leading to functional and aesthetic issues. This may occur in isolation, or as part of a polymalformative syndrome [6]. In addition to the morphological aspect, these clefts lead to disturbances in facial functions and growth (sucking, swallowing, phonation, abnormal bone bases) and can result in feeding, language and hearing disorders [8]. A multidisciplinary approach (maxillofacial and ENT surgery, speech therapy, orthodontics, psychology) is therefore essential [9]. Early cleft interventions such as lip-taping and nasoalveolar-molding can also be used during the neonatal period in order to reduce the severity of the deformity, even if the effectiveness of these techniques remains controversial [10]. Surgical management begins in the first few months of life, forming part of a multi-stage process spread over several years. The child’s course of treatment will, therefore, be marked by surgical interventions and hospitalizations, even if no real consensus has been reached regarding the age at which the various procedures should be performed or the surgical techniques that should be applied [11]. Several techniques are available for repairing orofacial clefts, depending on the type and extent of the cleft and the surgeon’s preferences, which are conditioned by the surgeon’s training, practice country, and structure [9]. The most commonly used techniques are the Millard (i.e., the “rule of 10s”) [12] and the Fisher [13] techniques for unilateral clefts, and the Millard technique for bilateral clefts [14]. Other techniques, such as the Mohler [15] or Noordhoff techniques [16], have also been described, and are frequently used in the United States [9]. In France, the most common surgical schedule for alveolar clefts is primary cheilo-rhinoplasty (lip closure) and intraveolar veloplasty (veil surgery) at the age of 6 months. If a residual cleft remains, it is closed at the age of 18 months. The alveolar cleft is closed at around 4 or 5 years old, after orthodontic treatment in temporary dentition, with the aim of restoring canine function and opening up space for the lateral incisor [17]. Depending on the case, gingivoperiostoplasty with alveolar bone grafting may be required, consisting of a cancellous bone graft taken from the child’s iliac crest and placed in the dental arch at the site of the cleft [18,19]. The aims of this procedure are manifold: not only does it re-establish continuity of the alveolar arch and close the mouth-to-nose communication, it also promotes eruption of the canine, enables better orthodontic treatment, and provides real support for the floor of the nasal cavity [20]. However, harvesting cancellous bone from the iliac crest presents potential per- and post-operative risks and complications, including pain, hematoma, and infection at the donor site [21]. In addition to the most common side effects, this procedure presents a risk of injury to the femoral–cutaneous nerve, damage to the epigastric vessels, parietal hernia or fracture of the iliac crest [22]. Finally, alveolar bone-grafting may prove insufficient due to excessive bone resorption from the autologous bone, necessitating further surgery. Indeed, the failure rate of these grafts is estimated at 20% [18].

Tissue engineering (TE), aiming to develop tissues or organs to restore, maintain or enhance biological functions, has undergone significant developments in recent years, and is opening up the field of regenerative medicine. This may eventually replace traditional organ transplants [23]. In the future, it could, therefore, replace bone autografts, making it possible to free oneself from the morbidity of the donor site and lower the age of cleft closure. To this end, numerous substitutes, such as homologous and xenografts, have been developed as an alternative to fill critical bone defects in oral and maxillofacial surgery [24,25,26]. On this topic, our team has developed an alginate-based hydrogel that provides an appropriate three-dimensional environment for transplanted cells, as well as a high angiogenic capacity and osteogenic potential [27]. Furthermore, the addition of human endothelial cells and/or mesenchymal stem cells (MSCs) to bone substitutes has now become an obvious way of improving the efficiency of bone regeneration [28]. MSCs are multipotent stromal cells that can differentiate into a variety of cell types, including osteoblasts—which are responsible for bone formation—and adipocytes—which are essential bone marrow components. To this end, a first study by our team focused on a honeycomb-shaped electrospun matrix composed of multiple layers of a synthetic polymer and hydroxyapatite nanoparticles, which are sometimes combined with MSCs. We evaluated its bone-regeneration capacity in vivo using a calvarial defect model in rats. Bone regeneration was studied by micro-computed tomography (μCT) over a 2-month period, and the data showed that the substitute alone improved the regeneration process, and that bone regeneration was more extensive, with newly formed bone being more mineralized, when combined with MSCs [29]. A second study was designed to evaluate the healing and osteogenic properties of the novel alginate-based hydrogel in a cleft palate model in rats. Hydrogel was seeded with bone-marrow-derived mesenchymal stem cells (BM-MSCs) or not, and incorporated into a surgically created cleft palate defect. Bone formation was assessed using μCT for 12 weeks, and the data showed that hydrogel alone did not differ significantly from the control group, but the addition of BM-MSCs stimulated bone formation at the margin of the defect and in the center of the implant [30]. Although the combination of bone substitutes and MSCs does not enable a full bone regeneration comparable to the healthy tissue at present, it still holds the potential to enhance the healing process, reduce the need for invasive surgeries, and thus improve patient outcomes.

Concerning in vivo models of a cleft lip and palate, numerous experimental models in different species have been described. Because of the congenital nature of this malformation, attempts have been made to induce it in utero by introducing a teratogenic treatment before closure of the bony palate [31,32]. However, the multifactorial origin of this pathology makes it difficult to develop a genetically modified model, and damage to a single gene can account for a wide variety of phenotypic expression, resulting in a lack of inter-individual reproducibility [33,34]. The surgical creation of an alveolar defect therefore seems the best compromise, and this procedure has been extensively explored, remaining the most widely used for the study of alveolar clefts as it is the most reproducible. The procedures for creating an alveolar defect are virtually identical in all species, whether primate [35], canine [36], swine [37], bovine [38], or rodent [30]. In all cases, and in order to be as close as possible to the human body, a form of mouth-to-nose communication must be created, and the defect must be large enough that spontaneous bone-healing alone cannot justify reconstruction [39]. In 2009, Nguyen et al. described a surgical technique that has since been widely adopted and optimized. After elevation of a mucoperiosteal gingival flap at the level of the incisivomolar diastema, a critical defect measurement of 7 × 4 × 3 mm was created using a ball burr [40]. Despite the critical size and, accordingly, the absence of ad integrum healing of the defect, spontaneous bone-healing remained a source of bias. With the aim of freeing ourselves from this, and to get as close as possible to human pathophysiology, we first wanted to optimize this model in order to implant the biomaterial in a site that cannot spontaneously heal, and whose bone healing can only be explained by the bone substitute.

The purpose of this study was therefore to evaluate the potential of a granular bone substitute using an optimized in vivo alveolar cleft model in rats. The bone substitute was placed into a surgically created bone defect in the rat’s jaw, with or without WJ-MSCs. The phenotype of the cells was analyzed using flow cytometry, and the expression of genes involved in osteoblastic and adipocytic differentiation was evaluated using reverse transcriptase PCR (RT-PCR). The bone-healing process was monitored using μCT to assess the volume and mineral density of the newly formed bone over time. This study validates our new optimized in vivo model of alveolar cleft in rats, and the data show significantly increased bone volume (BV) and bone mineral density (BMD) in the treatment groups compared with the control.

## 2. Materials and Methods

### 2.1. Cell Isolation and Culture

WJ-MSCs were isolated from umbilical cords obtained from three patients admitted to the Gynecology and Obstetrics Department of Amiens-Picardie University Hospital, as part of a biological collection authorized by the French Ministry of Education and Research under n°AC-2018-3320. The cords were stored in phosphate-buffered saline (PBS) (Sigma-Aldrich, Burlington, MA, USA), to which a mixture of Penicillin and Streptomycin (100 U/mL, 100 pg/mL, respectively) (Sigma-Aldrich) was added until they were used, up to 24 h after delivery. To collect Wharton’s Jelly, the two arteries and the umbilical vein were located and the WJ was stamped between them using cold blade number 15. 

Once cut into 1–2 mm sizes, fragments were placed 4 at a time in 6-well plates (4 fragments per well). Cells were cultured in Alpha Modified Eagle Medium (MEM) (Sigma-Aldrich) supplemented with 10% Fetal Calf Serum (FCS), 1% L-Glutamine and 1% Penicillin–Streptomycin (100 U/mL and 100 pg/mL, respectively) (1% PenStrep) (all Sigma-Aldrich). Cells were maintained in a humid atmosphere with 5% CO2, at 37 °C. Cord fragments were removed the 10th day and the medium was changed 2 times a week until confluence was reached.

At confluence, a first cell passage was performed, which consisted of removing the culture medium and rinsing the plates with 2 mL of PBS per well. The PBS was then removed, and the cells were detached and incubated for 5 min with 500 µL per well of trypsin–EDTA 0.025% (Sigma-Aldrich). Once the cells were detached, the trypsin–EDTA solution was neutralized with 1 mL of medium. Samples were collected and centrifuged at 1500 rpm for 5 min at 20 °C. After removal of the supernatant, the pellet was suspended in 2 mL of medium and the cells were reseeded in 2 T175 flasks. The culture medium was changed once a week until confluence. Cells were then counted and phenotyped before being frozen. A cryotube bank was created, with cells stored in a mixture of 10% DMSO/90% FCS at −150 °C.

### 2.2. Phenotypic Characterization

WJ-MSCs were characterized in comparison with BM-MSCs. The phenotype analysis was performed using flow cytometry (MACSQuantify, Miltenyi Biotec, Bergisch Gladbach, Germany). The cells were suspended, with a minimum of 100,000 cells per tube. The antibodies used at the correct concentration (depending on the antibodies) were added to the cell suspension (See Table A1). After incubation for 30 min in the dark at room temperature, the cells were rinsed with 1 mL of PBS and the excess antibodies were eliminated. The cells were suspended in 200 µL of MACS Buffer (MACSQuantify). A Fluorescence Minus One (FMO) control was performed for each of the panels.

### 2.3. Differientiation Capacities

To demonstrate the ability of WJ-MSCs to differentiate into osteoblasts and adipocytes, cells were placed under differentiation conditions for 21 days. Osteoblastic differentiation medium was composed of Dubellco’sMEM (DMEM) High Glucose (Sigma-Aldrich) supplemented with 10% FCS, 1% L-Glutamine, 1% PenStrep, and osteogenic factors consisting of 10 mM B-Glycerophosphate (Sigma-Aldrich), 50 mg/mL ascorbic acid (Sigma-Aldrich) and 10 nM Dexamethasone (Merck, Darmstadt, Germany). Adipocyte differentiation medium consisted of DMEM Low Glucose (Sigma-Aldrich) supplemented with 10% FCS, 1% L-Glutamine, 1% PenStrep^®^ and adipogenic factors consisting of 60 µM indomethacin (Sigma-Aldrich), 0.5 mM isobutylmethylxanthine (Sigma-Aldrich) and 10 nM Dexamethasone. Media were changed twice a week for 3 weeks.

At day 21, cells were stained with SIGMAFAST BCIP/NBT (Sigma-Aldrich) to reveal the Alkaline Phosphatase (ALP) enzymatic activity of osteoblasts, or with Red Oil (staining liposomes) for adipocytes. Cells were then observed under a microscope (Nikon Eclipse TS-100, Tokyo, Japan).

### 2.4. Biomaterial

For this study, the choice of biomaterial was synthetic biphasic calcium MaxResorb granules (60% HA; 40% phosphate-ß-tricalcium) (Straumann, Bâle, Switzerland), already marketed and used in maxillofacial and dental surgery [41]. Its dual composition enables two-stage resorption: the rapid degradation of ß-TCP frees up space for osteogenesis, while the slow resorption of HA maintains the inter-alveolar space. Granules range in size from 0.5 to 1 mm, with a nano-structured surface featuring interconnected macro- and micropores, and a porosity of around 80%, enabling cell adhesion, neo-angiogenesis and intercellular communication [42]. The cell-functionalized biomaterial was obtained using 12-well plates in which WJ-MSCs were cultured with the MaxResorb granules. Twenty-four hours prior to cell-seeding, the wells were filled with alphaMEM medium to buffer the acid pH. WJ-MSCs from three donors were used. After passage, cells were counted and reseeded at 50,000 cells per well in the presence of a proliferation medium, in order to have 1.6 million cells per well on the day of implantation.

### 2.5. Animals

**Figure 1:**
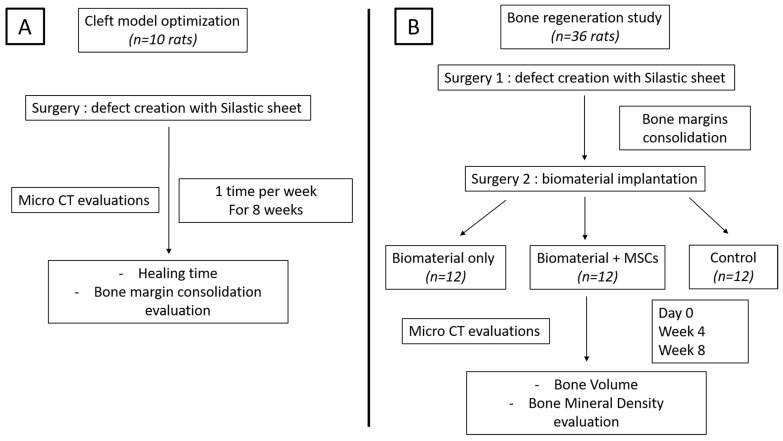
Study design: (**A**) cleft model optimization; (**B**) bone regeneration study.

All procedures were approved by the local ethics committee (Comité Régional d’Ethique en Matière d’Expérimentation Animale de Picardie (CREMEAP)) and the French Ministry of Research (reference APAFIS 31896-2021060415113703). Forty-six Sprague-Dawley rats, 8 weeks old (Janvier Labs, Le Genest-Saint-Isle, France), were housed in ventilated racks, under controlled conditions with ad libitum access to food and water. The model-optimization step required the use of 10 rats; then, the biomaterial implantation step required the use of 36 rats. A diagram of the study design is shown in Figure 1.

### 2.6. Surgical Procedures and Material Placement

**Figure 2:**
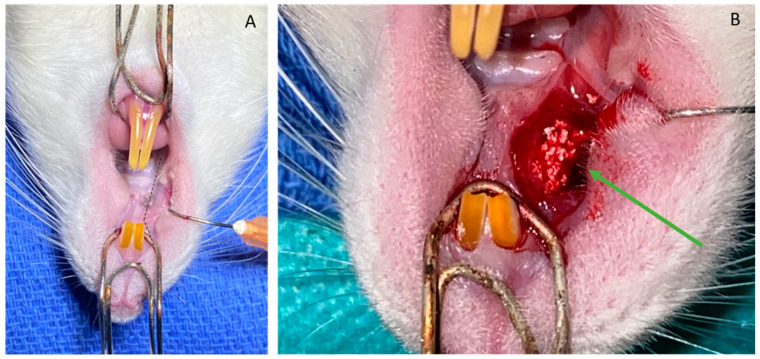
Intraoperative view at different stages of surgery: (**A**) installation and approach to the right maxilla (dots = incision line); (**B**) intraoperative view after creation of defect (green arrow).

All rats underwent the following procedure: under general anesthesia, the intraperitoneal injection of a mixture of ketamine (100 mg/kg) and xylazine (10 mg/kg) took place in the supine position, under apnea, after locating the right alveolar crest, as well as the subperiosteal infiltration of 0.3 mL of Lidocaine HCL 2%. An incision was made along the crest, from the right first molar to the neck of the right incisor (Figure 2A). Subperiosteal detachment with rugin took place, exposing the alveolar bone. Corticotomy was undertaken using a 0.8 mm diameter ball burr of 7 mm long, 3 mm high and 1 mm deep (Figure 2B). Placement of a silicone sheet (Silastic, Dow Chemical, Midland, MI, USA) conformed to the defect. A tight suture was made with separate stitches of Vicryl 5-0.

After a healing period of 5 weeks, the 36 rats in the second step underwent the following procedure: under general anesthesia, the intraperitoneal injection of a mixture of ketamine (90 mg/kg) and xylazine (6 mg/kg) was made in the supine position, under apnea, after locating the right alveolar crest, along with a subperiosteal infiltration of 0.3 mL of Lidocaine HCL 2%. The initial incision was reopened with a cold blade. Subperiosteal detachment with rugine took place. Silastic sheet was removed. Bone defect was filled with cellularized biomaterial (n = 12), biomaterial alone (n = 12) or left empty (n = 12). A tight suture was made with separate stitches of Vicryl 5-0.

An analgesic treatment was given systematically at the end of the procedure, and twice a day for 3 days, by subcutaneous injection of Buprenorphine 0.05 mg/kg. Daily monitoring with weighing and scoring was performed to ensure well-being and the absence of pain. Depending on the score, additional analgesic treatment could be provided by introducing Meloxicam 0.5 mg/mL into the drinking water or, if this was not sufficient, by a subcutaneous injection of Buprenorphine 0.05 mg/kg.

### 2.7. Tomodensitometric Analyses

Microtomographic analysis (µCT) was performed following each surgical procedure, under general anesthesia with isoflurane (induction with 5% isoflurane at an air flow rate of 1 L/min, and maintenance with 3% isoflurane at 0.5 L/min). Animals were scanned using a SKYSCAN 1176 machine (X-ray source: 65 kV, 380 μA, 1 mm Alu filter and 0.6 rotation pitch) (Bruker, Kontich, Belgium). Three-dimensional images were acquired with a maximum voxel size of 18 μm. The high-resolution, 3D, raw data set was obtained by rotating the flat panel detector 180° around the sample (scan time: 5 min). An internal density phantom (calibrated in grams per cubic centimeter of hydroxyapatite) was used to scale bone density.

During the model-optimization step, rats were scanned weekly for 8 weeks to determine the time required for the bone margins to heal. Following the implantation procedure, scans were taken 1 week before the second operation, just after implantation, and every 4 weeks for 8 weeks. Three-dimensional renderings were extracted from the data frames using DataViewers software (Bruker). A global grey value thresholding (55–255) was performed to separate mineralized elements from background noise. Defects and regenerated bone were measured using CT-scan analyzer software (Bruker). A global volume of interest (VOI) in the defect area was set by extrapolating 2D regions of interest over consecutive sections, including the remodeled bone defect area. The following parameters were analyzed: bone volume fraction (bone volume (BV)/tissue volume (TV), in %) and bone mineral density (BMD, in g/cm^3^ hydroxyapatite). These parameters were normalized to the volume of bone in the VOI prior to surgery. Three-dimensional images of the maxilla were reconstructed with CT vox (Bruker). The BV and BMD were used as indicators of the extent and quality of bone regeneration.

### 2.8. Statistical Analysis

The statistical analysis was performed using the Prism version 8 software (GraphPad Software, Boston, MA, USA). The data were expressed as mean ± standard deviation. The normality of the data was tested using the Shapiro–Wilk test. The comparison of the means was carried out using the Student’s *t*-test for paired data or the Mann–Whitney test for non-parametric data. The level of significance was set at *p* < 0.05.

## 3. Results

### 3.1. Cell Analysis

#### 3.1.1. Phenotype Analysis

**Figure 3:**
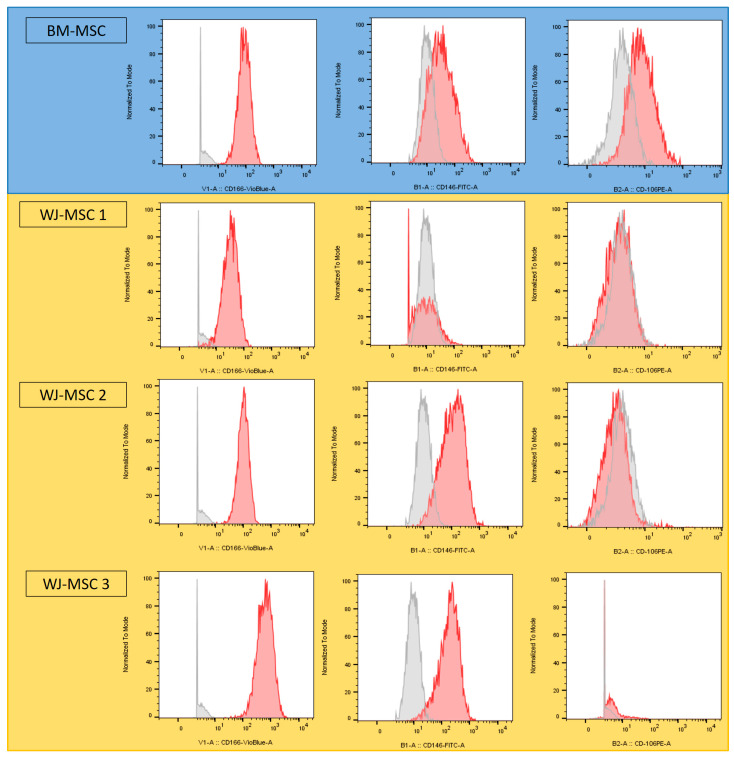
Cytometry results of CD106, CD146 and CD166 markers for BM-MSCs (blue) and WJ-MSCs (yellow). Grey spectra correspond to the Fluorescence Minus One (FMO) controls, and red spectra to the sample being analyzed.

Three Wharton’s jelly samples were collected from 3 different donors and analyzed against BM-MSC samples. Stem cell phenotypes were analyzed by flow cytometry prior to differentiation. All expressed the CD90, C73, CD105, and CD44 markers on their surface. CD19, CD39, CD34 and CD45 were not expressed (data not shown). Three cell adhesion proteins, (CD106, CD146 and CD166) were heterogeneously expressed depending on (i) the cell type and (ii) the donor. While BM-MSCs expressed all three proteins, all WJ-MSCs expressed CD166 on their surface, but only two expressed CD146 and none expressed CD106 (Figure 3).

#### 3.1.2. Differentiation Capacity Analysis

To characterize the capacity of WJ-MSCs to differentiate into osteoblasts and adipocytes, cells were cultured after amplification for 21 days in proliferation versus bone or adipocyte differentiation media. Osseous differentiation was studied with PAL staining and adipogenic capacity was studied with Red-Oil staining.

**Figure A1:**
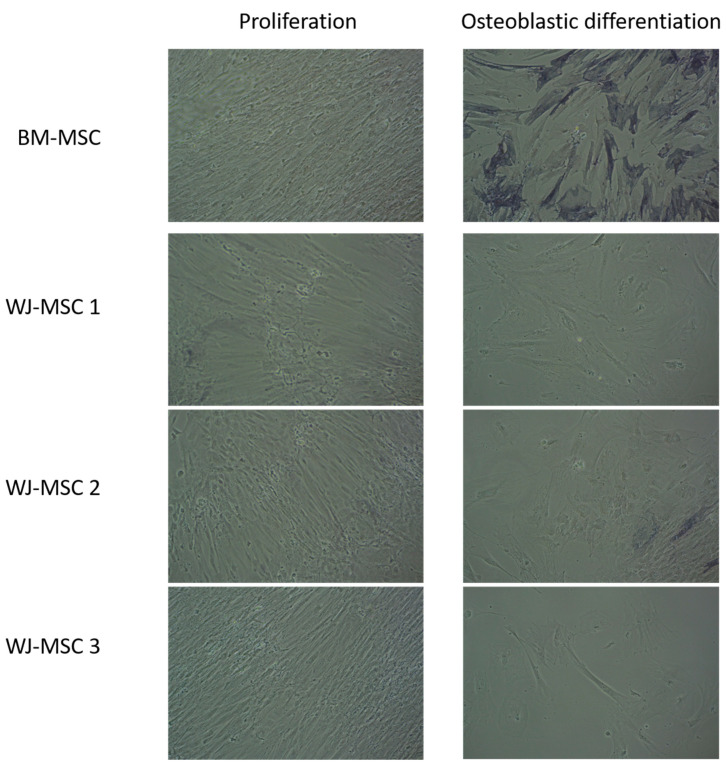
Microscopic observations (× 40) of PAL staining on BM-MSCs and WJ-MSCs in proliferation (control) and osteoblastic differentiation media.

The morphology of proliferating cells was fusiform with a centered nucleus, irrespective of their origin. In the osteoblastic condition, the 2 types of MSCs had the same morphology—different from the control condition—and appeared cuboid with extensions. However, WJ-MSCs appeared very slightly stained by PAL, unlike BM-MSCs (in line with the low PAL gene expression that was observed previously) (Figure A1).

**Figure A2:**
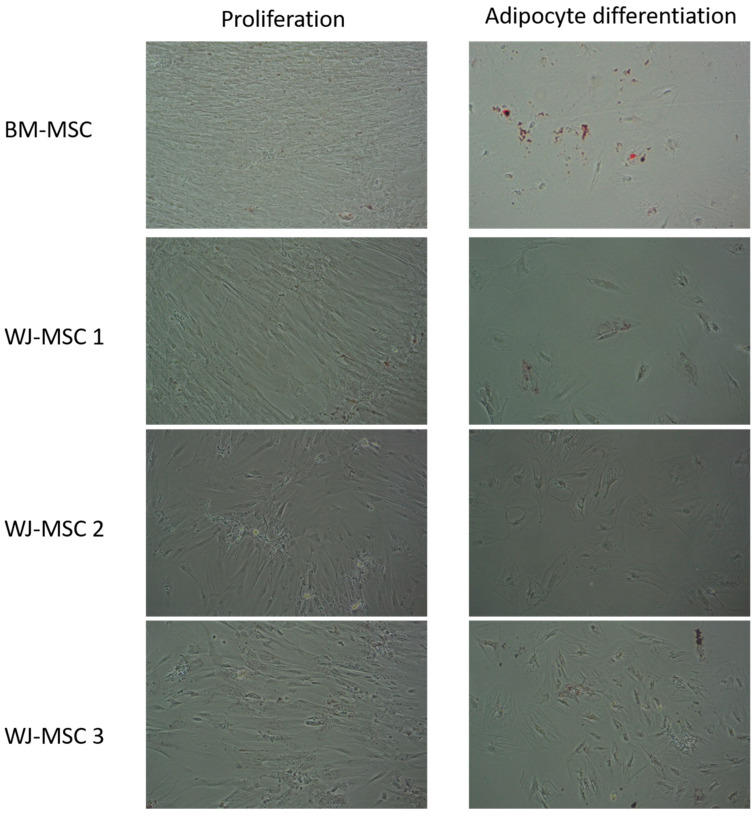
Microscopic observations (×40) of Red-Oil staining on BM-MSCs and WJ-MSCs in proliferation (control) and adipocyte differentiation media.

Similarly, cell morphology in the adipocyte condition appeared similar whatever the origin of the MSCs, and differed from the control condition: cells were increased in size, and polygonal in shape. Red-Oil staining revealed low liposome production in BM-MSCs, and no liposomes in WJ-MSCs. Despite morphological changes, the latter do not appear to be able to differentiate into adipocytes at 21 days under these conditions (Figure A2).

### 3.2. Cleft Model Optimization

#### 3.2.1. Surgical Procedure

This first step, which aimed to determine the healing time of the defect’s bone margins, enabled us to optimize the surgical procedure. Of the ten rats that were operated on, two died: one during anesthetic induction due to a probable drug overdose, the other postoperatively without revealing any particular cause. In order to avoid the death of the other animals, anesthetic dosages were reduced with the agreement of the local Structure for Animal Welfare. After a minor weight loss of under 10% during the first post-operative week, all animals regained their normal weight and continued to gain weight. Five out of eight rats showed porphyrin on the side of the incision during the operation and until euthanasia. The mucosal incision was initially wide, extending from the first premolar to the incisor, revealing the facial nerve after subperiosteal detachment. We then reduced the size of this incision to ensure respect for the facial nerve. A 1 mm ball-and-socket bur was initially used, before being replaced by a 0.8 mm one.

#### 3.2.2. Computed Tomography Analysis

Microtomographic analysis of the animals’ maxilla was performed weekly for 8 weeks. On average, the size of the defect was 6.4 ± 0.54 × 2.03 ± 0.1 × 2.17 ± 0.22 mm, with an average volume of 28.53 ± 4.69 mm^3^ (Table 1).

**Figure 4:**
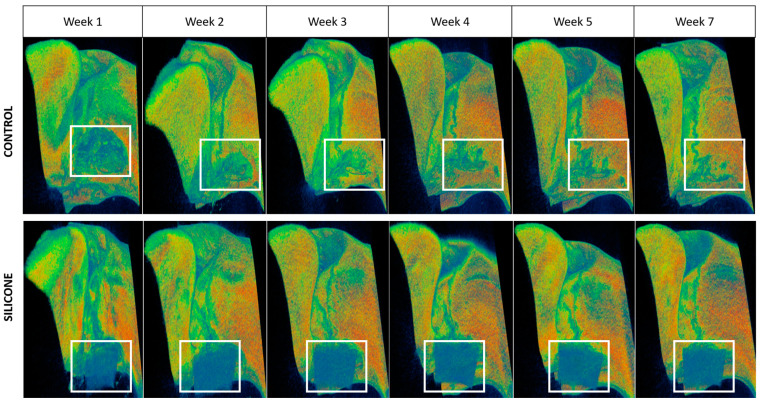
Representative microtomodensitometric images of bone healing over time (weeks 1, 2, 3, 4, 5 and 7) in the control and silicone conditions (area of interest framed in white, same rat per condition).

To better visualize the defect, a filter was added to color the voxels according to their density, with red corresponding to the highest densities (most mineralized), green to intermediate densities, and blue to low densities (poorly mineralized) (Figure 4). In all CT scans, densification of the bone margins was observed from the second week onwards, and became relevant from the 5th week. The FLAP1-2 rat lost its Silastic post-operatively at week 1 (not included in Table 1 and calculations). CT analysis showed spontaneous healing of the bone defect.

### 3.3. Bone Regeneration Study

#### 3.3.1. Biomaterial Implantation

Thirty-six rats underwent the initial surgical procedure to create a defect filled with a fragment of silicon plate. During this procedure, four rats died: 1 during anesthetic induction despite dose adjustment, 1 due to an intra-operative complication, and 2 did not wake up for no apparent reason. The post-operative course was identical to that of the optimization study, with weight loss during the first week followed by progressive weight regain in all rats. CT scans were performed at week 4. Loss of Silastic was observed in three rats and one Silastic had mobilization with endonasal passage. Defect dimensions were measured, with an average of 7.2 ± 0.65 × 2.43 ± 0.37 × 2.86 ± 0.68 mm (Table 2).

At week 5, the remaining 32 rats were operated on again. Two had mucosal wounds with Silastic exposure, and one died of probable inhalation. After resection of the former scar, the Silastic was easily removed, and the biomaterial—synthetic biphasic calcium MaxResorb granules, with or without WJ-MSCs—was inserted. Post-operative follow-up was identical to before, with initial weight loss followed by gradual weight regain.

#### 3.3.2. Computed Tomography Analysis

**Figure 5:**
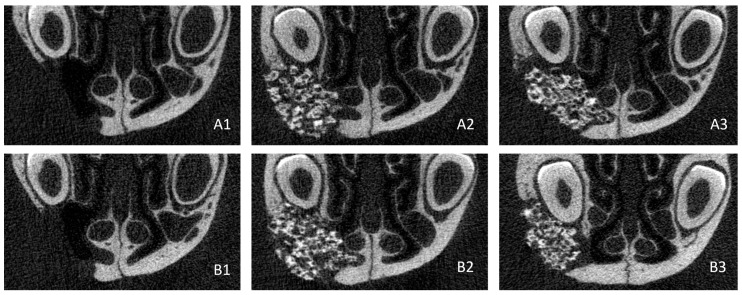
Transaxial CT sections at (**A**) week 4 and (**B**) week 8 under the following conditions: (1) empty, (2) biomaterial only and (3) biomaterial + MSCs.

Bone regeneration in the 3 groups was monitored by μCT immediately after surgery, at week 4, and at week 8. Visual analysis of the week 8 scans showed that bone-healing at the edges of the clefts was centripetal in all 3 groups, but that none had completely healed (Figure 5).

**Figure 6:**
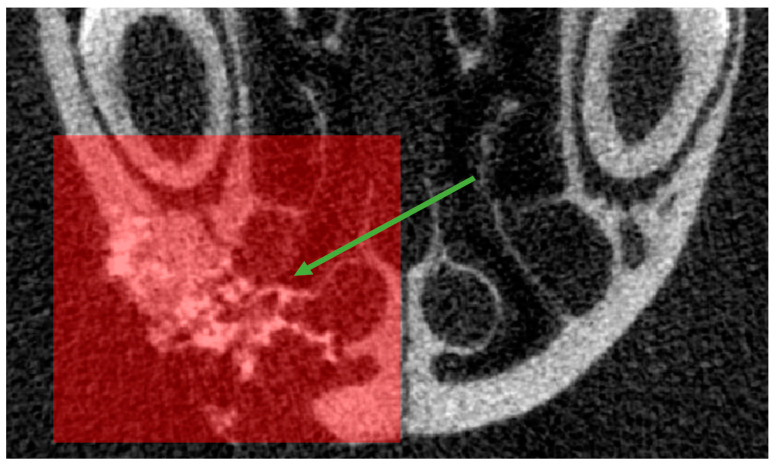
Transaxial scan section with selected VOI (red square). Note the thickening of the lateral wall and the filling of the aerial cavities (green arrow).

In order to accurately assess bone reconstruction, the volume of interest was plotted on the transaxial sections, from the most anterior region of the defect to the posterior, in a rectangular shape (Figure 6), thus obtaining a 3D VOI. The same VOI was applied to an un-operated rat of the same age as the control.

#### 3.3.3. Bone Volume and Bone Mineral Density

**Figure 7:**
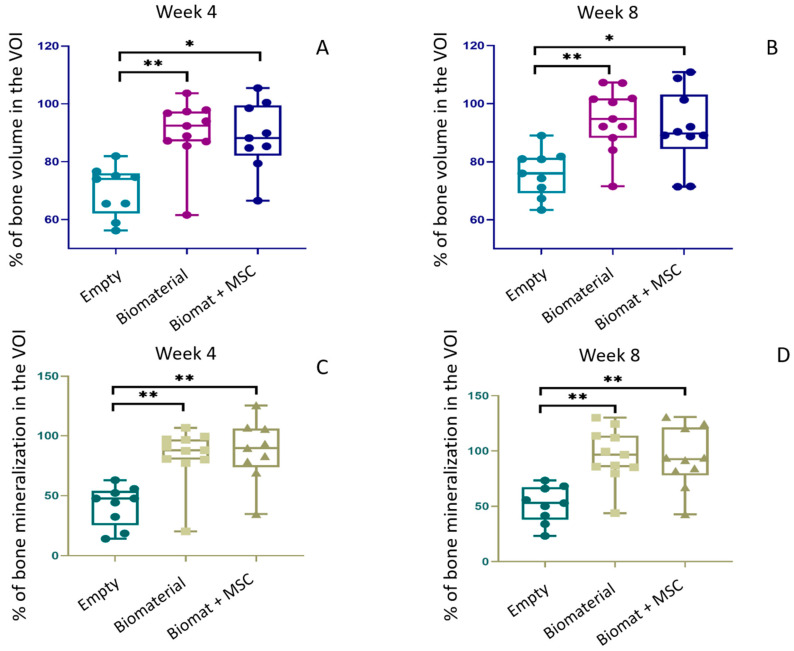
Bone volume (%) at week 4 (**A**) and week 8 (**B**) and bone mineralization (%) at week 4 (**C**) and week 8 (**D**) under “empty”, “biomaterial” and “biomaterial + MSC” conditions (* *p* ≤ 0.1; ** *p* ≤ 0.05).

In the “biomaterial only” and the “biomaterial + MSCs” groups, bone volume in the VOI increased significantly over time compared with the “empty” group, both at week 4, with 90.20% ± 11.03% and 88.73% ± 11.85%, vs. 69.84% ± 8.70% (Figure 7A), respectively, and at week 8, with 94.64% ± 10.71% and 91.33% ± 13.30%, vs. 76.09% ± 7.99% (Figure 7B), respectively. Interestingly, we reported no significant difference in BV for the “biomaterial + MSCs” group when compared to the “biomaterial only” group at either week 4 or week 8.

We also observed a significantly higher bone mineralization in the “biomaterial only” and “biomaterial + MSCs” groups compared with the “empty” group, both at week 4, with 84.09% ± 22.97% and 87.37% ± 26.02%, vs. 41.79% ± 16.63% (Figure 7C), respectively, and at week 8, with 96.13% ± 24.19% and 93.01% ± 27.04%, vs. 51.64% ± 16.51% (Figure 7D), respectively. Here, again, we found no significant difference in BMD between the two experimental conditions after 4 or 8 weeks.

All measured BV and BMD data are detailed in the Appendix section, in Table A2 and Table A3, respectively.

## 4. Discussion

In order to simulate the anatomy of a human alveolar cleft—with surrounding cortical bone margins—as closely as possible, we sought to optimize the rodent alveolar cleft model established by Nguyen et al. in 2009 [40]. We then introduced a fragment of a silicone plate into the surgically formed cleft, theoretically allowing for the bone margins to heal themselves. Regarding the size of the defect, we decided to produce a smaller defect than Nguyen et al.: although smaller, our defects are still of a critical size, in line with the study by Mostafa et al. (2014), who produced alveolar defects with a size of 5 × 2.5.1 mm [43]. Time to corticalization of the bone margins was estimated at 5 weeks post-op, after BMD measurement at the edges of the cleft. Densification of the bone margins in contact with the Silastic was observed in all rats with the silicone plate still in place, in contrast to the rat that lost it, which healed spontaneously. There are two possible explanations for the loss of Silastic: the first relates to the disunion of the endo-buccal scar, the second to mucosal erosion due to the silicone plate, resulting in its exposure and loss.

The difficulty of this surgery lies in fitting the Silastic, which must be adapted to fit precisely around the edges of the slot. Closure of the surgical site had to be precise to avoid any secondary mobilization of the plate. The silicone plate was visible on the CT scan, enabling us to monitor its position for 8 weeks. It should be noted that, in two rats, this caused slight bone resorption in the anterior part of the nasal septum, increasing the size of the defect. Moreover, during the second step, we noticed that some rats exposed part of their Silastic when the mucosa had healed, which could explain its loss. As a result, a consolidation time of longer than 5 weeks may not be recommended, as this would potentially increase the risk of losing the silicone plate. Three rats had lost theirs, so we had to reshape the newly formed bone during the second stage to re-establish the initial defect, thus inducing a bias in the analysis of bone reconstruction, as the defect edges were raw in these three rats.

Wharton’s Jelly MSCs were chosen for their ease of access and the absence of donor-site morbidity. Previous studies have shown them to have higher and faster proliferation, better immunomodulatory properties and a lower expression of the HLA-I system, making them less immunogenic that other MSC types [44]. Because of their pluripotency, WJ-MSCs can give rise to several cell types, including osteoblasts; however, their osteogenic commitment is lower than that of BM-MSCs, with longer delays in osteogenesis and a lack of expression of certain transcription factors [45]. Also, the extraction process of WJ-MSCs was described in the literature [46,47]. Our WJ-MSCs were characterized in comparison to BM-MSCs [48]. The results revealed a highly heterogeneous character with inter-individual variations in phenotypic expression, with a very limited capacity for differentiation into osteoblasts or adipocytes in vitro. These results are in line with the previous studies of our team [30] and the literature [49].

To implant the bone substitute during the second step of the study, the biomaterial proved difficult to get to grips with due to its presence in a well with little solidarity between the granules, whether or not they were cultured with WJ-MSCs. In addition, the placement of the material in the defect was also quite difficult due to the restricted access to the defect and its small size compared with the size of the granules. Thus, the manufacturing of the substitute should be improved in further studies, for example, by either (i) adding a hydrogel to ensure that the granules and MSCs are held together and/or (ii) by greatly increasing the number of MSCs that are used.

The bone regeneration in the three groups was followed by μCT immediately after the operation, after 4 weeks, and after 8 weeks. The scans were analyzed to assess the newly formed bone volume and mineral density within the defect. In order to analyze the reconstructed bone volume, we had to select a VOI. We focused on a rectangular volume on transaxial sections, taking in (i) the defect, (ii) the granules and (iii) the defect environment. In scans, we observed a densification of the dental organ on the operated side, associated with a thickening of the lateral surface along the dental root. The VOI therefore corresponds to a wide zone that does not only take in the defect, causing a loss of precision and potentially explaining results beyond 50% bone, even under “empty” conditions. However, our results show that both BV and BMD significantly increased in the experimental groups compared with the control group at 4 and 8 weeks. They also show that, under “biomaterial only” and “biomaterial + MSCs” conditions, three rats rebuilt more than 100% of their bone at week 4, compared to control. Similarly, after 8 weeks, eight rats exceeded 100% bone reconstruction. These results may be explained by a change in the operated side, with air cavities being filled by granules or being ossified, increasing overall bone volume on the operated side compared with the non-operated rat. In order to consider these biases, a new VOI could be defined, considerig only the created defect. However, this would be difficult due to the complex geometry of the created lesion. The results would be more representative of bone regeneration specific to the defect, but would no longer take into account changes in the graft area’s environment.

Moreover, we observed spontaneous bone healing over time in the “empty” condition, with an increase in bone volume despite the Silastic plate being in place for 5 weeks. However, this reconstruction was small enough to show the effect of the calcium granules +/− MSCs on bone regeneration. This spontaneous healing is a specific bias of our rat model, which we may have freed ourselves from by using a larger animal model, such as a pig [28]. Even if spontaneous healing persists despite the optimization of our model, the bone substitute placement in the cleft after 5 weeks did not result in an enlargement of the bone defect, as was the case in the previous study by Naudot et al. [30].

In this study, we did not notice a significant difference between the “biomaterial only” and “biomaterial + MSCs” conditions, whereas previous studies by our team show a significant increase in bone regeneration when MSCs are added [29,30]. This may be due to a too-small number of initially implanted cells since we used 50,000 cells, compared with 1,000,000 in the two previous studies. This choice was made on the basis that, unlike previously, we pre-cultured WJ-MSCs on biomaterial for 15 days, and we were counting on the cells’ doubling time to obtain 1.6 million MSCs at the time of implantation. However, due to the limited number of bone substitutes that were created, we were unable to verify the number of present cells prior to implantation, although we know that this number is a key factor in bone regeneration. This will therefore be the focus of particular attention in our future work.

In the end, this pilot study provides valuable insights into the regenerative capabilities of a bone substitute made of biphasic calcium granules, using an optimized alveolar cleft model in rats. The biomaterial results showed BV and BMD improvements compared to the control group, with and without MSCs. Further studies are now needed to optimize the manufacturing of the bone substitute including biphasic calcium granules and MSCs to potentially target the treatment of alveolar clefts in clinical practice.

## 5. Conclusions

The optimization of a previously published alveolar cleft model in rats enabled us to graft our bone substitute in an environment that was as close as possible to that of a real patient. The use of a silicone sheet made it possible to control the spontaneous regeneration associated with rats, and the first step of our study led us to evaluate the time to consolidation of bone margins at 5 weeks. The results of the second step of our study using this optimized in vivo model are promising concerning both the newly formed bone volume and bone mineral density. Our data show a significant increase in these two parameters under the two experimental conditions (biomaterial composed of biphasic calcium granules alone, and biomaterial with the addition of WJ-MSCs) when compared to the control “empty” condition. However, neither treatment resulted in total healing of the defect, and further studies are now essential to optimize the bone substitute manufacturing before considering it for human use in the long term. This pilot study thus provides a foundation for future research in the field of tissue engineering applied to the treatment of alveolar clefts, and contributes to the understanding of the regenerative capabilities of bone substitutes in a relevant animal model.

## Figures and Tables

**Table 1:** Measurement of defects in 7 rats after 8 weeks.

ID	Length (mm)	Width (mm)	Height (mm)
FLAP 1-02	6.600	1.970	2.333
FLAP 1-03	7.053	2.146	2.322
FLAP 1-04	6.139	1.987	1.811
FLAP 1-06	5.664	2.181	2.216
FLAP 1-07	6.103	1.899	2.286
FLAP 1-08	7.194	2.005	2.321
FLAP 1-09	6.402	2.040	1.900
Mean	6.450	2.033	2.170
SD	0.544	0.100	0.220

**Table 2:** Measurement of defects in 30 rats (with Silastic still in place) at week 4.

ID	Length (mm)	Width (mm)	Height (mm)
FLAP 2-01	7.2	2.1	2.4
FLAP 2-02	6.4	2.1	2.6
FLAP 2-04	6.9	2.9	2.2
FLAP 2-05	7.1	2.24	3.3
FLAP 2-06	7.4	2.2	2.1
FLAP 2-07	6.1	2.3	2.1
FLAP 2-08	7.7	2.3	3.4
FLAP 2-09	6.8	2	3
FLAP 2-10	6.3	2.7	4.3
FLAP 2-11	7.1	2.7	4.8
FLAP 2-12	8.6	2.3	3.1
FLAP 2-13	7.1	2.8	2.6
FLAP 2-14	8.1	3.1	3.6
FLAP 2-15	7.4	2.6	2.9
FLAP 2-16	7.3	2.3	3.5
FLAP 2-17	7.7	3.1	3.4
FLAP 2-18	7.5	3.1	3.6
FLAP 2-20	6.5	2	2.7
FLAP 2-21	6.8	2	2.4
FLAP 2-22	6.9	2.1	3
FLAP 2-25	6.4	2	2
FLAP 2-26	8.4	2.4	2.3
FLAP 2-27	7.8	2.4	2.5
FLAP 2-29	7	2.4	2
FLAP 2-30	7.6	2.3	2.5
FLAP 2-31	6.6	2.2	2.1
FLAP 2-33	6.6	2.3	3.2
FLAP 2-34	7.1	3.1	3
FLAP 2-35	7.2	2.9	2.8
FLAP 2-36	8.5	2.1	2.4
Mean	7.203	2.435	2.860
SD	0.648	0.370	0.679

## Data Availability

Data are contained within this article, main text or Appendix A.

## Appendix A

**Table A1:** List of antibodies and panels used.

Antibody			Miltenyi Biotec Reference			Panel		
CD44-BV2011			562,890			1		
CD90-FITC			559,869			1		
CD34-PE			555,822			1		
CD73-PECy7			561,258			1		
CD105-APC			562,408			1		
CD45-APCH7			557,833			1		
CD166-BV421			562,936			2		
CD146-FITC			560,846			2		
CD106-PE			555,647			2		
CD39-APCH7			560,239			2		
Channel	V1	V2	B1	B2	B3	B4	R1	R2
Panel 1	CD44	Viability	CD90	CD34		CD73	CD105	CD45
Panel 2	CD166	Viability	CD146	CD106		CD19	CD39	

**Table A2:** Bone volume fraction (BV/TV) in Volume of Interest at week 4 and week 8 (%).

	Empty		Biomaterial		Biomaterial + MSCs	
	Week 4	Week 8	Week 4	Week 8	Week 4	Week 8
	65.4870226	71.1397164	103.679512	107.26423	84.7664404	88.7551674
	58.872241	63.4156236	88.8578814	92.1241866	79.3924439	90.3266916
	75.1626814	80.8667322	61.5975858	71.5300296	66.5312816	71.420468
	81.914415	88.9777144	87.2144574	92.1070676	89.8781738	92.0591344
	56.1777104	67.3358746	92.3843954	88.2689878	88.2039356	89.1180902
	65.5554986	75.9980886	86.9713676	84.0474424	85.3176722	71.5300296
	74.673078	81.794582	97.8727468	101.584146	100.457716	89.1317854
	74.0328274	74.29646	94.000429	101.827236	98.5301164	108.753583
	76.6451868	80.9454796	97.355753	100.440597	105.463311	101.337632
	N/A	N/A	85.492286	94.7742078	N/A	110.845525
	N/A	N/A	96.7771308	107.099888	N/A	N/A
Mean	69.836	76.086	90.200	94.643	88.727	91.328
SD	8.701	7.985	11.026	10.713	11.845	13.295

**Table A3:** Bone mineral density (BMD) in Volume of Interest at week 4 and week 8 (%).

	Empty		Biomaterial		Biomaterial + MSCs	
	Week 4	Week 8	Week 4	Week 8	Week 4	Week 8
	18.74994	34.0908	87.49972	85.79518	82.95428	84.09064
	47.72712	23.29538	80.68156	85.227	69.31796	93.7497
	52.27256	65.90888	20.45448	43.74986	34.65898	42.6135
	63.06798	73.29522	88.0679	96.5906	93.18152	93.7497
	14.2045	41.47714	96.5906	86.93154	89.77244	91.47698
	32.38626	55.68164	80.11338	79.5452	78.40884	67.04524
	55.68164	68.1816	97.15878	130.11322	106.81784	81.81792
	47.72712	49.99984	90.9088	113.636	105.68148	124.43142
	44.31804	52.84074	106.81784	111.93146	125.56778	120.45416
	N/A	N/A	77.27248	99.4315	N/A	130.6814
	N/A	N/A	99.4315	124.43142	N/A	N/A
Mean	41.793	51.641	84.091	96.126	87.373	93.011
SD	16.631	16.513	22.970	24.191	26.020	27.043
